# Is Ciprofloxacin a Substrate of P-glycoprotein?

**DOI:** 10.1111/j.1753-5174.2010.00032.x

**Published:** 2011-03

**Authors:** Miki Susanto Park, Hideaki Okochi, Leslie Z Benet

**Affiliations:** * Department of Pharmaceutics and Medicinal Chemistry, Thomas J. Long School of Pharmacy and Health Sciences, University of the PacificStockton, CA, USA; † Department of Bioengineering and Therapeutic Sciences, University of CaliforniaSan Francisco, CA, USA

**Keywords:** Ciprofloxacin, P-glycoprotein, MRP1, MRP2

## Abstract

**Introduction:**

Studies using MDCKII and LLC-PK1 cells transfected with MDR1 cDNA indicate that ciprofloxacin is not a substrate of P-glycoprotein. However, our data has shown that transport studies done using different P-gp overexpressing cell lines (MDCKI-MDR1, MDCKII-MDR1 and L-MDR1), could lead to contradictory conclusion on whether a compound is a substrate of P-gp. The aim of our study was to determine if ciprofloxacin is indeed not a P-glycoprotein substrate using MDCKI cells transfected with human MDR1 cDNA.

**Methods:**

Semi-quantitative RT-PCR was used to determine the mRNA level of MDR1 while Western blot was performed to determine the protein expression level of P-gp, MRP1 and MRP2 in various cells. Ciprofloxacin bidirectional transport studies were performed in MDCKI, MDCKI-MDR1, MDCKII, MDCKII-MDR1, MDCKII-MRP2, LLC-PK1, L-MRP1 and L-MDR1 cells.

**Results:**

Ciprofloxacin showed net secretion in MDCKI-MDR1 but net absorption in MDCKI cells. Various P-gp inhibitors decreased the B to A and increased the A to B transport of ciprofloxacin in MDCKI-MDR1 cells while having no effect in MDCKI cells. The B to A transport of ciprofloxacin in MDCKI-MDR1 cells was not affected by non-P-gp inhibitors. In the presence of indomethacin, ciprofloxacin showed net secretion instead of net absorption in MDCKI cells while in the presence of probenecid and sulfinpyrazone, there was no net secretion and absorption. There was no difference in ciprofloxacin transport between MDCKII and MDCKII-MDR1, LLC-PK1 and L-MDR1, LLC-PK1 and L-MRP1 and MDCKII and MDCKII-MRP2.

**Conclusions:**

Transport data in MDCKI and MDCKI-MDR1 cells indicate that ciprofloxacin is a substrate of P-gp but data from MDCKII, MDCKII-MDR1, LLC-PK1 and L-MDR1 cells indicate that ciprofloxacin is not a substrate of P-gp. Vinblastine, a well-known P-gp substrate, also did not show differences between LLC-PK1 and L-MDR1 cells. Further studies need to be performed to characterize these P-gp overexpressing cell lines and the transport of ciprofloxacin.

## Introduction

Ciprofloxacin is an antibiotic belonging to the quinolone family with a broad spectrum antibactericidal activity [1]. The oral bioavailability of ciprofloxacin is 50–80% [2] and at least 10% of ciprofloxacin is eliminated via intestinal secretion [3], of which <1% is due to biliary excretion [4]. A number of studies have reported that intestinal secretion of ciprofloxacin does not seem to be mediated by P-glycoprotein (P-gp) [5–9]. Using LLC-PK1 cells transfected with the human MDR1 cDNA (L-MDR1), de Lange et al. [7] showed that there was no difference between the apical to basal (A to B) and basal to apical (B to A) transport of ciprofloxacin in L-MDR1 cells. However, ciprofloxacin did significantly inhibit the transport of rhodamine-123, a known P-gp substrate, in L-MDR1 cells [7]. Lowes and Simmons [9] showed that there was no net secretion of ciprofloxacin in MDCKII and MDCKII-MDR1 cells, suggesting that ciprofloxacin is not a P-gp substrate.

The aim of this study was to determine whether ciprofloxacin is indeed not a P-gp substrate using MDCKI cells transfected with human MDR1 cDNA (MDCKI-MDR1) [10]. Our data has shown that transport studies done using different P-gp overexpressing cell lines, MDCKI-MDR1, MDCKII-MDR1 [11] and L-MDR1 [11], could lead to contradictory conclusion on whether a compound is a substrate of P-gp. For example, vinblastine is a widely accepted P-gp substrate and transport studies done in our lab in MDCKI-MDR1 cells confirm that it is a substrate of P-gp. However, studies done using LLC-PK1 and L-MDR1 cells showed no difference in vinblastine transport between those two cell lines, which would suggest that vinblastine is not a P-gp substrate [12]. Another example is trimethoprim, shown to be a substrate of P-gp based on studies in MDCKI-MDR1 and L-MDR1 cells [13]. However, there was no difference in trimethoprim transport between MDCKII and MDCKII-MDR1 cells [12].

## Methods

### Cell Culture

MDCKI, MDCKI-MDR1 (a gift from Dr. Ira Pastan of the National Institutes of Health), MDCKII, MDCKII-MDR1 and MDCKII-MRP2 (a gift from Prof. Dr. Piet Borst of the Dutch Cancer Institute) were cultured in Dulbecco's modified Eagle's medium (DMEM) supplemented with 10% fetal bovine serum (FBS) while LLC-PK1, L-MRP1 and L-MDR1 cells (a gift from Prof. Dr. Piet Borst of the Dutch Cancer Institute) were cultured in M-199 media with 10% FBS. For all the cells that contained the MDR1 cDNA, 80 ng/mL colchicine was added to the media to select for the transfectant cells. Cells were seeded onto PET cell culture inserts of a 6-well plate system at a density of roughly 300,000 cells/insert and grown to confluency as a monolayer for 5–7 days at 37°C and 5% humidified CO_2_-atmosphere. The media was changed every 2–3 days.

### Bidirectional Transport Study

The transport experiments were adapted with modifications from Zhang et al. [14]. Most experiments were repeated at least twice and there were triplicates in each study. To determine if ciprofloxacin is a substrate of P-gp, MRP1 or MRP2, bidirectional transport studies were performed in the controls and P-gp, MRP1 or MRP2 overexpressing cell lines. All cells were fed fresh media the day before the transport studies. On the day of the experiments, the cells were washed once and preincubated for ∼15 minutes at 37°C in 5% CO_2_ with Hank's Balanced Salt Solution containing 22.5 mM HEPES (HBSS-H). To measure ciprofloxacin transport in the B→A direction, 2.5 mL of HBSS-H solution containing the drug was put into the basal (B) side and 1.5 mL of HBSS-H was put into the apical (A) side. At selected time points, 200 µl aliquot were taken from the A side and replaced with fresh HBSS-H. For measuring ciprofloxacin transport in the A→B direction, the drug solution was put into the A side and aliquots were taken from the B side. For inhibition studies, the inhibitor was put in both the A and B sides. During the studies, the cells were incubated in a shaking incubator at 37°C. To establish cell integrity, [^14^C]-mannitol (a paracellular marker) (NEN, Boston, MA) transport was measured for 1 hour at the end of the experiments. Transport of [^3^H]-digoxin (NEN, Boston, MA) and [^3^H]-vinblastine (Amersham, Piscataway, NJ), known P-gp substrates, was also measured as positive controls.

### Sample Analysis

Samples were stored at −20°C until analysis by high performance liquid chromatography (HPLC) using a Zorbax SB-C18 250 × 4.6 mm (Phenomenex, Torrance, CA) column. The mobile phase was methanol and 0.5% glacial acetic acid (50:50, v/v). The flow rate was 1 mL/min. An UV detector (Agilent, Santa Clara, CA) was used to detect ciprofloxacin at 280 nm.

### Western blot

To compare P-gp, MRP1 and MRP2 expression in our cell lines, Western blot and RT-PCR were performed. For Western blot, cells were grown in T75 flasks, rinsed with PBS Ca^2+^, Mg^2+^-free solution and scraped into 15 ml Falcon tubes. The tubes were centrifuged for 15 minutes at 6000 g at 4°C. The supernatants were discarded and the pellets were washed again with PBS Ca^2+^, Mg^2+^-free solution and centrifuged. The resulting pellets were resuspended with lysis buffer pH 7.4 (10 mM KCl, 10 mM Tris-HCl, and 1.5 mM MgCl_2_), put on ice and sonicated for 20 s (3×). The protein concentrations were determined by BioRad assay. All samples were diluted to the same concentration and mixed with Laemmli sample buffer (1:3), loaded onto 7.5% SDS-PAGE gels and run at 200 V for ∼45 minutes. The gels were incubated with blotting buffer (25 mM Tris, 192 mM glycine, 20% methanol) for 15 minutes at 4°C, then blotted to nitrocellulose membranes for 1 hour at 200 mA. The membranes were blocked with 5% dry milk solution in Tris-buffered solution (TBS) for 2 hours at room temperature (RT) and then washed with TBS containing 0.05% Tween-20 (TTBS). Depending on the proteins of interest, the membranes were incubated overnight at 4°C with either 50x diluted anti-MRP1 clone MRPr1 antibody (MC-201, Kamiya Biomedical, Seattle, WA), 500x diluted c219 (for detecting P-gp) antibody (Signet, Dedham, MA) or 50x diluted anti-MRP2 clone M2 III-6 (MC-206, Kamiya Biomedical, Seattle, WA). The next day, the membranes were washed several times with TTBS before incubation for 1 hour at RT with their appropriate secondary antibody (3000x diluted goat anti-mouse (Gibco BRL Life Technologies, Grand Island, NY) for c219 and anti-MRP2 antibodies and goat anti-rat (Boehringer-Ingelheim, Indianapolis, IN) for anti-MRP1 antibody. The membranes were washed several times with TTBS and incubated with premix enhanced chemiluminescent reagents 1 & 2 (Amersham, Piscataway, NJ) for 1 minute before developing the film.

### One Step Semi-quantitative RT-PCR

The cells were rinsed in PBS Ca^2+^ Mg^2+^ free solution and RNA isolation was done according to the protocol outlined in TRIzol (Invitrogen Life Technologies, Carlsbad, CA). The quantity of extracted RNA was determined by spectrophotometric analysis. The RNA samples were stored at −80°C until RT-PCR. One step cDNA synthesis and PCR reaction was done using the Qiagen OneStep RT-PCR Reaction Kit (Qiagen, Valencia, CA) in PCR Express machine (Thermo Hybaid, Ashford, Middlesex, United Kingdom). Sequences of the primers used were as follows: forward primer: 5′-GCC TGG CAG CTG GAA GAC AAA TAC ACA AAA T-3′, and reverse primer: 5′-AGA CAG CAG CTG ACA GTC CAA GAA CAG GAC T-3′ (Invitrogen Life Technologies, Carlsbad, CA). This primer pair produced a 285 bp segment of the *MDR1* gene. For internal standard, we utilized the *18S* gene with the competimer technology from Ambion (Austin, TX). The expected product of the 18S gene was 489 bp. The cycling parameters were as follows: cDNA synthesis at 50°C for 30 minutes; denaturation step for 15 minutes at 95°C; amplification step (26 cycles) was 1min at 94°C _ 1 minute at 59°C _ 1 minute at 72°C; extension step was 15 minutes at 72°C. The RT-PCR products were run on 2% E-gel (Invitrogen, Carlsbad, CA) for 30 minutes at 66 V. The bands were visualized with the UV transilluminator and a picture was taken with a Polaroid camera.

### Data Analysis

For the bidirectional transport study experiments, the total amount of drugs transported into the other side was calculated according to the following formula:


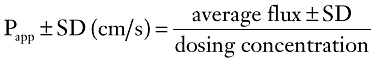


Drug flux was calculated using the LINEST function from Microsoft Excel. The LINEST function calculated the slope of the line that best fits the amount of drugs transported *vs.* time plot. Flux values were calculated for each of the triplicates and were averaged to give us the average value of the flux and the standard deviation associated with it. The apparent permeability value was calculated as follows:


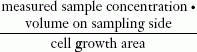


Statistical significance was tested by ANOVA using the Primer Express program created by Dr. Stanton Glantz (UCSF, San Francisco, CA).

## Results and Discussion

### Characterization of Cell Lines

Before conducting each transport study, cell integrity and monolayer confluency were confirmed by microscopy and transepithelial electrical resistance (TEER) measurements. Under the microscope, P-gp, MRP1 and MRP2 overexpressing cell lines (i.e. MDCKI-MDR1, MDCKII-MDR1, MDCKII-MRP2, L-MRP1, and L-MDR1) appeared to have different morphologies compared to their respective control cell lines. MDCKI-MDR1 and L-MRP1 cells grew faster than their control cell lines, MDCKI and LLC-PK1 cells, respectively. They also had higher TEER values. The average TEER values for MDCKI, MDCKI-MDR1, LLC-PK1 and L-MRP1 were 150, 1500, 165 and 240 Ω respectively. However, TEER value measurement was not very accurate and precise. There were large variations associated with the values. Furthermore, for many cell lines such as MDCKI, MDCKII, MDCKII-MDR1, LLC-PK1 and L-MDR1, TEER values were very close to the values measured in the absence of any cells (∼140 Ω). Therefore, to further monitor cell integrity and monolayer confluency, at the end of the experiments, we also measured [^14^C]-mannitol (a paracellular marker) transport for 1 hour. As expected, we did not find any significant difference in the B to A and A to B transport of mannitol in our cell lines (data not shown). Unlike TEER values, we also did not find large variations in the P_app_ of mannitol between controls and overexpressing cell lines. For example, there was about a 10-fold difference in TEER values between MDCKI and MDCKI-MDR1 cells (∼150 and 1500 Ω respectively) but the P_app_ values for mannitol in those two cell lines were approximately the same (∼5 × 10^−7^cm/s). The mannitol P_app_ values in MDCKII, MDCKII-MRP2 and MDCKII-MDR1 cells were similar to MDCKI and MDCKI-MDR1 cells. They were higher in LLC-PK1, L-MDR1 and L-MRP1 cells (∼13 × 10^−7^, 12 × 10^−7^ and 35 × 10^−7^cm/s, respectively).

**Figure 1:**
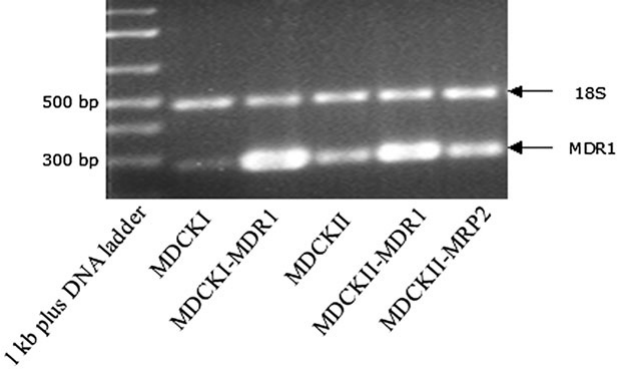
RT-PCR results of MDR1 mRNA comparison in several cell lines.

We also performed Western blot and RT-PCR studies to compare the expression of Pgp/*MDR1*, MRP1 and MRP2 among our cell lines. Figure 1 shows the result of an RT-PCR experiment to compare the expression of MDR1 mRNA among various cell lines. As expected, expression of *MDR1* mRNA was much higher in P-gp overexpressing cell lines, MDCKI-MDR1 and MDCKII-MDR1 cells, compared to their control cell lines, MDCKI and MDCKII cells, respectively. The results also show that the expression of *MDR1* mRNA was higher in MDCKII cells compared to MDCKI cells.

**Figure 2:**
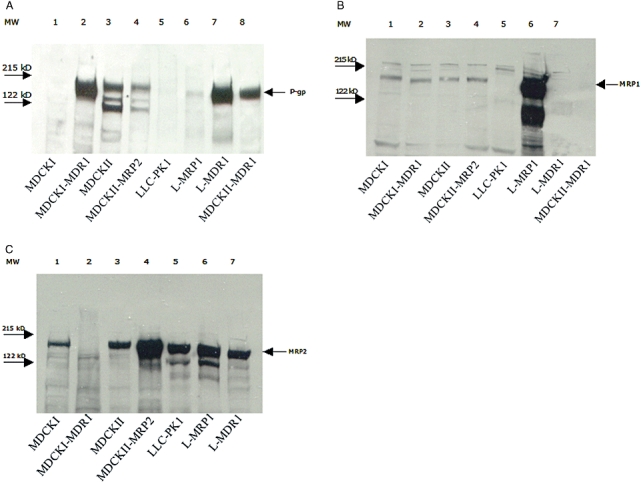
Western blot results of (A) P-gp; (B) MRP1; and (C) MRP2 expression comparison among various cell lines.

Figure 2A shows the Western blot result of P-gp (∼170 kDa) expression comparison among various cell lines. Western blot result agrees with the observations from RT-PCR studies (Figure 1). P-gp expression was higher in P-gp overexpressing cell lines, MDCKI-MDR1, MDCKII-MDR1 and L-MDR1 cells, compared to their control cell lines, MDCKI, MDCKII and LLC-PK1 cells, respectively. The result also shows that P-gp expression was higher in MDCKII compared to MDCKI or LLC-PK1 cells.

Figures 2B and C show the Western blot result of MRP1 and MRP2 (∼190 kDa) expression comparison among various cell lines, respectively. As expected, MRP1 expression was much higher in the MRP1 overexpressing cell line, L-MRP1 cells, compared to its control cell line, LLC-PK1 cells, where MRP1 expression was not observed. The identity of the abundant lower band is not known but we think it could be the unglycosylated form of MRP1 protein. The result also shows that MRP1 expression was similar in MDCKI, MDCKI-MDR1, MDCKII and MDCKII-MDR1 cells. MRP2 expression was much higher in the MRP2 overexpressing cell line, MDCKII-MRP2 cells, compared to its control cell line, MDCKII cells. It is interesting that no MRP2 expression was detected in a P-gp overexpressing cell line, MDCKI-MDR1 cells, which suggests that MRP2 expression was downregulated in that cell line, versus its control, MDCKI cells. However, no downregulation of MRP2 expression was observed in another P-gp overexpressing cell line, the L-MDR1 cells.

### Bidirectional Transport Study Data

**Figure 3:**
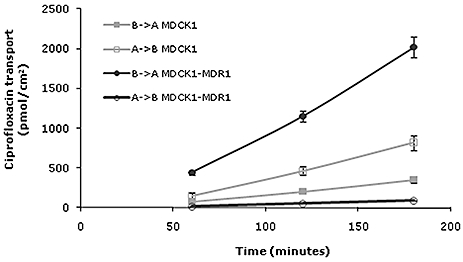
Bidirectional transport of 100 µM ciprofloxacin in MDCK1 (gray line) and MDCK1-MDR1 (black line) cells. 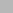, • = B→A and □, ○ = A→B.

Because P-gp is an efflux transporter that pumps drugs out from cells into the apical solution, for a P-gp substrate, the B to A flux should be greater than the A to B, with the difference more pronounced in the P-gp overexpressing cell line. For ciprofloxacin, the B to A flux was higher than the A to B flux in the MDCKI-MDR1 cells, with the B to A flux higher and A to B flux lower compared to MDCKI cells (Figure 3). The B to A/A to B ratio was about 10 in MDCKI-MDR1 cells and it was abolished to about 1 at 4°C (Table 1). This indicates that ciprofloxacin is a substrate of P-gp. Interestingly, the B to A flux was lower than the A to B flux in MDCKI cells. MDCKI cells have an endogenous expression of P-gp, as shown by our RT-PCR studies (Figure 1). Normally for a P-gp substrate, e.g., digoxin and vinblastine, the B to A flux is higher or equal to the A to B flux in MDCKI cells. But it was the opposite for ciprofloxacin. The A to B flux was higher than the B to A flux, with the B to A/A to B ratio of about 0.4 in MDCKI cells (Table 1). This result agrees with the data from Cavet et al. [5]. This suggests that ciprofloxacin, besides being a substrate of P-gp, is also a substrate of an absorptive transporter that pumps in the opposite direction of P-gp. We believe it is an energy dependent transporter because at 4°C, the difference between the A to B and B to A fluxes in MDCKI cells was abolished, bringing the B to A/A to B ratio up from 0.4 to about 1 (Table 1).

**Table 1:** Bidirectional transport of 25 and 100 µM ciprofloxacin in MDCKI and MDCKI-MDR1 cells

		P_app_ × 10^−7^ (avg ± SD, n = 3, cm/s)		B→A
			
Cell Line	Concentration	B→A	A→B	A→B
MDCKI	25	4.5 (0.4)	10.6 (1.2)	0.4
MDCKI	100	3.8 (0.4)	9.2 (0.9)	0.4
MDCKI 4°C	25	2.1 (0.7)	1.7 (0.4)	1.2
MDCKI-MDR1	25	35.4 (1.0)	3.5 (0.4)	10
MDCKI-MDR1	100	21.9 (1.4)	1.0 (0.03)	22
MDCKI-MDR1 4°C	25	1.3 (0.4)	1.1 (0.2)	1.2
No cells	25	128.3 (2.8	153.7 (30.9)	0.8

To further test if ciprofloxacin is a P-gp substrate, we investigated the effect of various inhibitors on ciprofloxacin transport in MDCKI and MDCKI-MDR1 cells. P-gp inhibitors impede drug efflux from the cells out into the apical side, therefore, in the presence of P-gp inhibitors, the B to A flux will be decreased and the A to B will be increased. If complete inhibition of P-gp function is achieved and no other transporters are involved, the B to A flux should equal the A to B flux. The effect of P-gp inhibitors will be more pronounced on P-gp overexpressing than control cell lines since the P-gp overexpressing cell line has a greater flux difference between the B to A and A to B directions.

**Figure 4:**
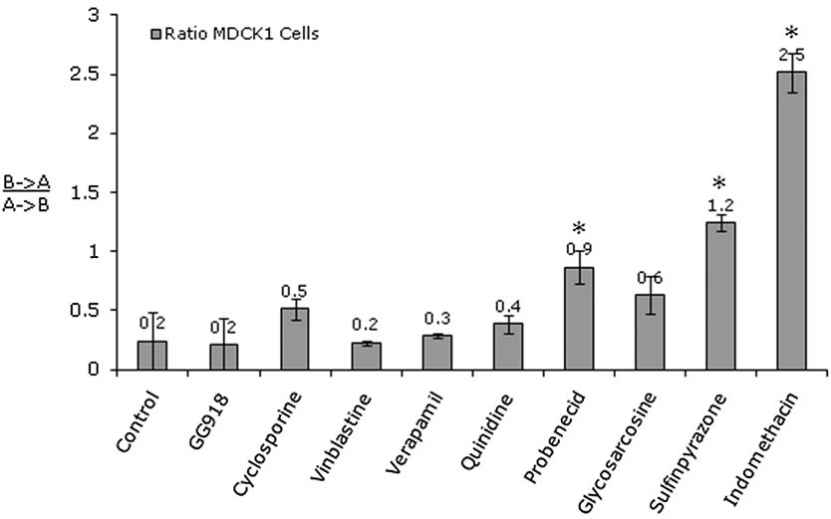
Effect of various inhibitors on 25 µM ciprofloxacin B→A/A→B ratio in MDCK1 cells. The inhibitors are GG918 (2.5 µM), cyclosporine (20 µM), vinblastine (100 µM), verapamil (50 µM), quinidine (100 µM), probenecid (100 µM), glycosarcosine (10 mM), sulfinpyrazone (1 mM) and indomethacin (100 µM). *P < 0.01.

GG918, cyclosporine, ketoconazole, vinblastine, verapamil and quinidine are P-gp inhibitors. Dicloxacillin, trimethoprim and erythromycin are P-gp substrates. Glycosarcosine is an inhibitor of PEPT1, a pH-dependent uptake transporter. Probenecid and PAH are inhibitors of organic anion transporters. TEA is an inhibitor of organic cation transporters. Sulfinpyrazone and indomethacin are MRP1 and MRP2 inhibitors. Figure 4 shows the effect of various inhibitors on ciprofloxacin B to A/A to B ratio in MDCKI cells. In this study, in the absence of any inhibitors, the B to A/A to B ratio of ciprofloxacin was about 0.2. The ratio increased significantly to about 0.9, 1.2 and 2.5 with probenecid, sulfinpyrazone and indomethacin, respectively. P-gp inhibitors (GG918, cyclosporine, vinblastine, verapamil, and quinidine) and a PEPT1 inhibitor (glycosarcosine) had no significant effects on ciprofloxacin transport in MDCKI cells. It is interesting that we did not observe any effects with P-gp inhibitors. We expected the B to A/A to B ratio to decrease with P-gp inhibitors. One explanation could be that the ratio was low and it is hard to reduce something that was already low. Another explanation could be that those inhibitors, besides inhibiting P-gp activity, could also inhibit the activity of the unidentified absorptive transporter.

The fact that glycosarcosine had no effect implies that ciprofloxacin is not a substrate of PEPT1. We had confirmed this further by running a pH gradient comparison study, where we did not see any difference (data not shown). It is interesting that in the presence of a MRP1 and MRP2 inhibitor, indomethacin, the B to A/A to B ratio flipped from less than 1 to about 2.5. This suggests that indomethacin inhibited the activity of the unidentified absorptive transporter, and when this transporter was inhibited, P-gp effect could be observed. The data also suggest that MRP1 could be the unidentified absorptive transporter but studies using L-MRP1 and MDCKII-MRP2 indicate that ciprofloxacin is not a substrate of MRP1 and MRP2 (Tables 2 and 3). The absorptive transporter could be an organic anion transporter since probenecid increased the B to A/A to B ratio of ciprofloxacin. Vanwert et al. [15] has shown that ciprofloxacin is a substrate of OAT3 and probenecid is an inhibitor of OAT3. Probenecid was also shown to competitively inhibit ciprofloxacin renal secretion in humans [16].

**Table 2:** Bidirectional transport of 25 µM ciprofloxacin in MDCKII, MDCKII-MRP2 and MDCKII-MDR1 cells

	P_app_ × 10^−7^ (avg ± SD, n = 3, cm/s)		B→A
		
Cell Line	B→A	A→B	A→B
MDCKII	17.6 (3.6)	15.6 (2.5)	1.1
MDCKII-MRP2	15.0 (1.1)	11.3 (7.3)	1.3
MDCKII-MDR1	6.8 (1.2)	7.5 (1.2)	0.9

**Table 3:** Bidirectional transport of 25 µM ciprofloxacin in LLC-PK1, L-MRP1 and L-MDR1 cells

	P_app_ × 10^−7^ (avg ± SD, n = 3, cm/s)		B→A
		
Cell Line	B→A	A→B	A→B
LLC-PK1	23 (2.3)	18 (1.8)	1.3
L-MRP1	17.5 (0.9)	19.6 (1.0)	0.9
L-MDR1	22.6 (1.7)	12.8 (1.8)	1.8

**Figure 5:**
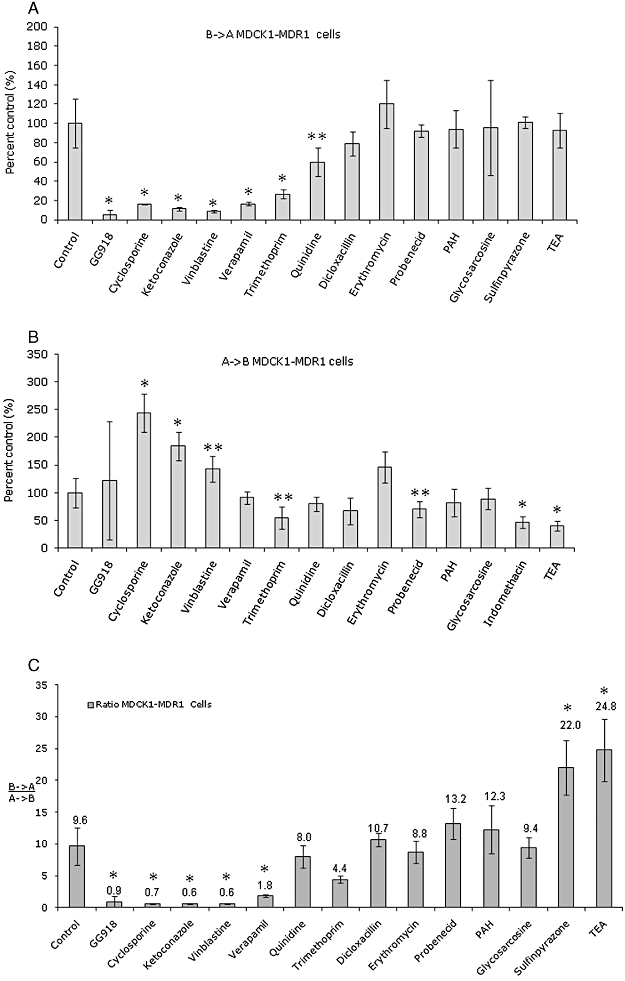
Effect of various inhibitors on 25 µM ciprofloxacin (A) B→A transport; (B) A→B transport and (C) B→A/A→B ratio in MDCK1-MDR1 cells. The inhibitors are GG918 (2.5 µM),cyclosporine (20 µM),ketoconazole (100 µM), vinblastine (100 µM), verapamil (50 µM), trimethoprim (100 µM), quinidine (100 µM), dicloxacillin (100 µM), erythromycin (100 µM), probenecid (100 µM), PAH (100 µM), glycosarcosine (10 mM), sulfinpyrazone (1 mM) and TEA (100 µM). *P < 0.01 **P < 0.05.

In MDCK1-MDR1 cells, the B to A flux significantly decreased in the presence of P-gp inhibitors (GG918, cyclosporine, ketoconazole, vinblastine, verapamil, trimethoprim, quinidine) while non-P-gp inhibitors (probenecid, PAH, glycosarcosine, sulfinpyrazone, TEA) had no effect (Figure 5A). As expected with P-gp inhibitors, the A to B flux significantly increased with cyclosporine, ketoconazole and vinblastine (Figure 5B). Trimethoprim, probenecid, indomethacin and TEA decreased the A to B flux. This could mean that they inhibited the activity of the unidentified absorptive transporter. Figure 5C shows the effect of various inhibitors on ciprofloxacin B to A/A to B ratios in MDCK1-MDR1 cells. As expected, P-gp inhibitors decreased the ratio while sulfinpyrazone and TEA increased the ratio, which is presumed to be due to inhibition of the function of the unidentified absorptive transporter.

**Figure 6:**
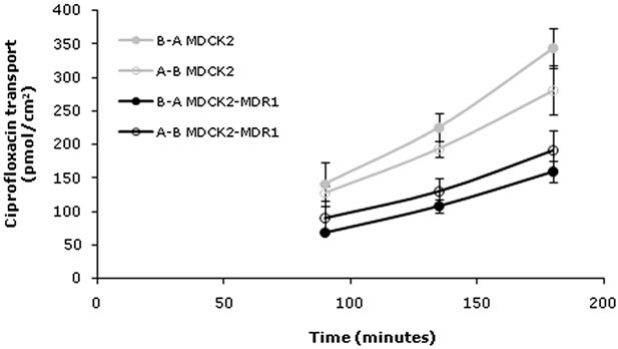
Bidirectional transport of 25 μm ciprofloxacin in MDCKII (gray line) and MDCKII-MDR1 (black line) cells. 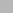, • = B→A and □, ○ = A→B.

In MDCKII cells, the ciprofloxacin B to A flux was almost equal to the A to B flux (Figure 6). This was not what we observed in MDCKI cells. Western blot and RT-PCR studies show that P-gp expression was higher in MDCKII compared to MDCKI cells (Figures 1 and 2A). Therefore, we believe that in MDCKI cells, which had lower expression of P-gp, the absorptive transporter played a bigger role than P-gp, which then resulted in higher A to B than B to A fluxes for ciprofloxacin. In MDCKII cells, where the P-gp expression was higher, the two transporters contribute to the same extent, with equal B to A and A to B fluxes, therefore we did not see the difference between the B to A and A to B fluxes in this cell line. This will hold true only if ciprofloxacin is a substrate of canine P-gp. The B to A and A to B fluxes were lower in MDCKII-MDR1 compared to MDCKII cells (Figure 6). However, there was no difference between the B to A and A to B flux for ciprofloxacin in MDCKII-MDR1 cells, with the B to A/A to B ratio of about 1 (Table 2), suggesting that ciprofloxacin is not a substrate of P-gp. This agrees with the data reported by Lowes and Simmons [9]. As reported by de Lange [7], there was also no difference in the B to A fluxes of ciprofloxacin between LLC-PK1 and L-MDR1 cells but the A to B flux was slightly lower in L-MDR1 cells but the difference was not statistically significant (Table 3).

Currently we do not know the cause of the discrepancy among these P-gp overexpressing cell lines. The endogenous expression of other proteins could be affected depending on the method used to create P-gp overexpressing cell lines. The Western blot data show that MRP2 expression was downregulated in MDCKI-MDR1 cells but not in L-MDR1 cells (Figure 2C). It is possible that besides P-gp, the expression of another transporter was upregulated in MDCKI-MDR1 (but not in MDCKII and L-MDR1) cells and this transporter was responsible for the observed ciprofloxacin B to A/A to B difference between MDCKI and MDCKI-MDR1 cells and the activity of this transporter in MDCKI-MDR1 cells was inhibited by the P-gp inhibitors used in our studies. Further studies need to be performed to determine if ciprofloxacin is indeed not a P-glycoprotein substrate. One such study could be a comparison in brain accumulation of ciprofloxacin between wild type and P-gp knockout mice, mdr1a/b (-/-) mice. No statistically significance difference in ciprofloxacin accumulation in brains of wild type and P-gp knockout mice would indicate that ciprofloxacin is not a substrate of P-gp. However, this cannot conclusively show that ciprofloxacin is not a substrate of human P-gp since species difference in P-gp transport activity has been reported [17].
